# Defining carotid near-occlusion with full collapse: a pooled analysis

**DOI:** 10.1007/s00234-021-02728-5

**Published:** 2021-05-04

**Authors:** Elias Johansson, Thomas Gu, Allan J. Fox

**Affiliations:** 1 Clinical Science, Neurosciences, Umeå University, 90182 Umeå, Sweden; 2 Wallenberg Centre for Molecular Medicine, Umeå University, 90182 Umeå, Sweden; 3 Sunnybrook Health Science Centre, 2075 Bayview Ave, Toronto, ON M4N 3M5 Canada

**Keywords:** Stroke, Carotid stenosis, Carotid near-occlusion, CT-angiography, Ultrasound

## Abstract

**Purpose:**

Create a new definition of near-occlusion with full collapse to predicting recurrent stroke.

**Methods:**

Pooled analysis of two studies. Patients with symptomatic ≥ 50% carotid stenoses were included. Outcome was preoperative recurrent ipsilateral ischemic stroke or retinal artery occlusion within 28 days of presenting event. We analyzed several artery diameters on computed tomography angiography and stenosis velocity on ultrasound.

**Results:**

A total of 430 patients with symptomatic ≥ 50% carotid stenosis were included, 27% had near-occlusion. By traditional definition, 27% with full collapse and 11% without full collapse reached the outcome (*p* = 0.047). Distal internal carotid artery (ICA) diameter, ICA ratio, and ICA-to-external carotid artery ratio were associated with the outcome. Best new definition of full collapse was distal ICA diameter ≤ 2.0 mm and/or ICA ratio ≤ 0.42. With this new definition, 36% with full collapse and 4% without full collapse reached the outcome (*p* < 0.001).

**Conclusions:**

Defining near-occlusion with full collapse as distal ICA diameter ≤ 2.0 mm and/or ICA ratio ≤ 0.42 seems to yield better prognostic discrimination than the traditional appearance-based definition. This novel definition can be used in prognostic and treatment studies of near-occlusion with full collapse.

## Introduction

**Fig. 1:**
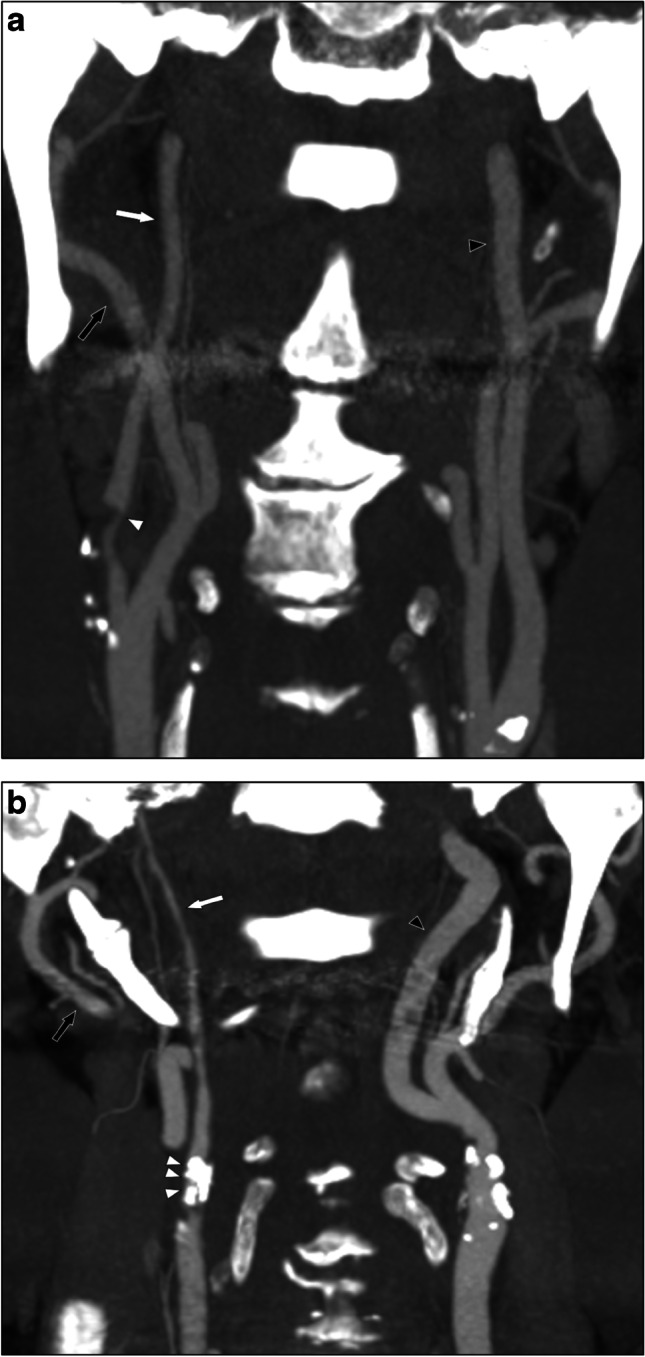
Carotid near-occlusions, coronal views. **a** Right-sided near-occlusion without full collapse. Beyond a severe stenosis (white arrowhead), the distal ICA is normal-appearing (white arrow, diameter 2.8 mm). However, the distal ICA is smaller than contralateral ICA (black arrowhead, ICA ratio 0.72) and similar to ipsilateral ECA (black arrow, ECA ratio 0.97). It was classified as without full collapse with all presented definitions. **b** Right-sided near-occlusion with full collapse. Beyond a severe and calcified stenosis (white arrowheads), the distal ICA has a threadlike appearance (white arrow, diameter 1.2 mm), clearly smaller than both contralateral ICA (black arrowhead, ICA ratio 0.20) and ipsilateral ECA (black arrow, ECA ratio 0.40). It was classified as with full collapse with all presented definitions

Carotid near-occlusion is a severe carotid stenosis where the internal carotid artery (ICA) distal to the stenosis is reduced in size (“collapsed”) [1–3]. The distal ICA size reduction is often subtle, resulting in normal-appearing but small distal ICA (near-occlusion without full collapse, Fig. 1a). The distal ICA size reduction can be severe, resulting in a threadlike distal ICA (near-occlusion with full collapse, Fig. 1b). Conventional carotid stenoses do not cause distal ICA size reduction [1].

Guidelines recommend conservative treatment of symptomatic near-occlusion based on trials where 94% of near-occlusion were without full collapse [1–5]. Two studies have shown that near-occlusion with full collapse has a high risk of recurrent ipsilateral ischemic stroke during the first days after the presenting event, but the risk for near-occlusion without full collapse was modest [6, 7]. One study reported recurrent stroke in 18% of near-occlusion with full collapse, but with unclear timing and no control group [8]. However, one registry study found a low early risk for all near-occlusions [9]. As a high, possibly treatable, risk of stroke seems to exist for near-occlusion with full collapse, the definition of full collapse becomes relevant to establish. The traditional definition of full collapse, a threadlike distal ICA appearance, might not be optimal for prognosis. No other definition has been compared with the risk of recurrent stroke. A measurement-based definition, either anatomical (artery size) or physiological (flow velocity), might not only have better prognostic discrimination, but will likely be more reliable between observers than the traditional definition.

## Aim

The aim of this study was to create a new definition of near-occlusion with full collapse based on angiographic and/or ultrasound measurements, aimed at predicting preoperative recurrent ipsilateral ischemic stroke.

## Methods

This was a pooled individual patient data analysis of two previously reported studies [6, 7]. The design of the two studies is summarized in Table 1. Both included patients with symptomatic ≥ 50% (NASCET) carotid stenosis that were eligible for revascularization. Symptomatic stenosis was defined as < 6 months since last ipsilateral event (a vast majority was within days). Eligible for revascularization was defined as either undergoing revascularization or were reasonable candidates for revascularization. Reasonable candidate were cases with issues such as recurrent severe stroke, patient refusal, technical considerations, and when risks outweighed benefit after careful consideration. However, cases with very advanced age, severe co-morbidity, and severe index stroke were excluded. In this analysis, only those that were examined with computed tomography angiography (CTA) of sufficient quality within 6 months of the presenting event were included. Ultrasound, which was otherwise used alone, has poor sensitivity for near-occlusion [10, 11]. A total of 430 patients with symptomatic ≥ 50% carotid stenosis were included of which 116 (27%) had near-occlusion, 47 with and 69 without full collapse. Baseline characteristics are presented in Table 2. The included studies had ethical approval as presented in their original articles [6, 7].

**Table 1:** Design summary of the two included studies

	Johansson et al. [6]	Gu et al. [7]
Years on inclusion	2007–2009	2010–2014
Site	Umeå, Sweden	Umeå, Sweden
Main purpose of original study	Assess risk of recurrent stroke in all ≥ 50% stenosis	Assess risk of recurrent stroke in near-occlusion
Design	Prospective	Retrospective
Case detection approach	Diagnosis of ≥ 50% stenosis in clinic routine	Re-assessment of 4440 consecutive CTAs
Total cases with symptomatic ≥ 50% stenosis	230	514
Cases excluded due to no relevant CTA (% of total)	164 (71)	149 (29)
Included cases (% of total)	66 (29)	365 (71)
Conventional ≥ 50% stenosis *n* (%)	49 (74)	266 (73)
Near-occlusion without full collapse^a^ *n* (%)	12 (18)	57 (16)
Near-occlusion with full collapse^a^ *n* (%)	5 (8)	42 (12)

*CTA* computed tomography angiography

^a^Division by traditional definition

**Table 2:** Baseline comparisons between stenosis groups, using traditional definition of full collapse

		Missing data	Conventional ≥ 50% stenosis (*n* = 315)	Near-occlusion without full collapse (*n* = 69)	Near-occlusion with full collapse (*n* = 47)	*p*^a^
Age mean^b^ (SD)		0	72 (8)	70 (9)	70 (8)	0.02
Women^b^ *n* (%)		0	96 (30)	19 (28)	16 (34)	0.77
Myocardial infarction^b^ *n* (%)		1	65 (21)	12 (17)	6 (13)	0.40
Symptomatic peripheral artery disease^b^ *n* (%)		1	25 (8)	8 (12)	3 (6)	0.56
Current angina^b^ *n* (%)		2	50 (16)	7 (10)	6 (13)	0.48
Heart failure^b^ *n* (%)		2	21 (7)	3 (4)	2 (4)	0.68
Current smoker^b^ *n* (%)		3	51 (16)	16 (23)	9 (19)	0.39
Diabetes^b^ *n* (%)		1	78 (25)	18 (26)	9 (19)	0.68
Hypertension^†^ *n* (%)		1	289 (92)	56 (81)	42 (89)	0.02
Previous arterial revascularization^b,c^ *n* (%)		1	68 (22)	11 (16)	9 (19)	0.54
Type of presenting event	Afx	0	33 (10)	13 (20)	3 (6)	0.16
	RAO		16 (5)	2 (3)	1 (2)	
	TIA		117 (37)	28 (41)	15 (32)	
	Stroke		149 (47)	26 (38)	28 (60)	
Sought medical attention on day of presenting event n (%)		0	232 (74)	44 (64)	34 (72)	0.26
Delay presenting event—CTA median (IQR)		0	3 (1–7)	5 (2–15)	4 (1–14)	0.03
Delay presenting event—ultrasound median (IQR)		93^d^	6 (3–12)	7 (4–16)	9 (5–18)	0.15
Underwent revascularization^e^ *n* (%)		0	209 (66)	55 (78)	13 (28)	< 0.001
Delay presenting event—revascularization median (IQR)		0	10 (7–21)	13 (7–24)	18 (9–29)	0.12
Stenosis diameter mm mean (SD)		11^f^	1.4 (0.5)	0.8 (0.3)	0.7 (0.3)	< 0.001
Distal ICA diameter mm mean (SD)		0	4.2 (0.6)	2.9 (0.5)	1.1 (0.8)	< 0.001
ICA-ratio mean (SD)		16^ g^	1.00 (0.26)	0.64 (0.11)	0.30 (0.28)	< 0.001
ECA ration mean (SD)		0	1.64 (0.40)	1.08 (0.47)	0.40 (0.29)	< 0.001
Stenosis PSV m/s mean (SD)		93^d^	2.9 (1.4)	3.7 (1.7)	2.2 (2.4)	< 0.001

*Afx* amaurosis fugax, *CTA* computed tomography angiography, *ECA* external carotid artery, *ICA* internal carotid artery, *IQR* interquartile range, *PSV* peak systolic velocity, *RAO* retinal artery occlusion, *SD* standard deviation, *TIA* transient ischemic attack

^a^*χ*^2^-test, one-way ANOVA, and Kruskal–Wallis

^b^Used as co-variate in the step-wise removal multivariable analysis

^c^Coronary, carotid, or leg artery interventions

^d^84 had no ultrasound exam and 9 had echo shadow. 31 were among near-occlusions

^e^Carotid endarterectomy or angioplasty with stenting

^f^7 were among near-occlusions, caused by severe calcification

^g^4 were among near-occlusions, caused by contralateral occlusion

### Outcomes

The outcome was defined the same way as in both underlying studies and major trials [3, 6, 7]: recurrent ipsilateral ischemic stroke or ipsilateral retinal artery occlusion (RAO). Stroke was defined by the World Health Organization definition [12], i.e., required symptoms to last > 24 h, whereas transient ischemic attack (TIA, not part of main outcome) was symptoms lasting < 24 h, regardless of imaging evidence of fresh ischemia. RAO was defined as monocular vision loss lasting > 24 h, whereas amaurosis fugax (not part of outcome) lasted < 24 h. In both studies, only anterior circulation events were considered. The risk of the outcome was assessed during the first 28 days after presenting event, until revascularization or death, whichever came first.

### Parameters

Both previously published and unpublished data were available for analysis. Available data included baseline factors, recurrent events, CTA assessments, CTA measurements, and velocity data from carotid ultrasound. In Gu et al., CTAs were performed locally or at any of 11 referring hospitals, in Johansson et al., only the referring hospital. In both, various machines and clinical protocols were used for CTA. In both studies, ultrasound was performed at the local hospital. The ultrasound exams were performed by several experienced sonographers using a standardized protocol including long- and short-axis assessment of common, internal, and external carotid arteries, with color doppler, pulsed doppler in duplex mode, and power doppler. Main emphasis was assessment of highest peak systolic velocity (PSV) in the stenosis, with adjustable angle correction ≤ 60°. Various ultrasound machines were used during the studies.

In Gu et al., near-occlusion was diagnosed on CTA by two observers (EJ, 5 years carotid grading experience and AF, > 40 years carotid grading experience) using an approach of systematic interpretation of features, not over interpreting anatomical variance as near-occlusion [7, 13]. Cases without near-occlusion (conventional stenosis) were graded by comparing smallest stenosis lumen diameter with the distal ICA diameter. Per NASCET method, the distal ICA was measured well beyond the bulb as the ICA is usually larger in the bulb region [3]. For this analysis, the same two observers reassessed the CTAs from Johansson et al. with the same approach. In both studies, the two observers were blinded to each other and to the outcome.

Five parameters were candidates for novel definitions of full collapse. Four were CTA-based: stenosis diameter, distal ICA diameter, ipsilateral to contralateral distal ICA diameter ratio (ICA ratio), and distal ICA to ipsilateral external carotid artery diameter ratio (ECA ratio). Stenosis PSV was based on ultrasound. CTA measurements were gathered by one observer (EJ) with a random selection of 49 cases measured twice for reliability analyses. Stenosis PSVs were extracted from reassessment of images by several overlapping observers. All measurement extraction was done blinded to the outcome.

### Analyses and statistics

Individual patient data were assessed in a pooled database. The analysis was conducted in four steps. (1) We assessed if any of the five candidate parameters was significantly associated with the outcome among near-occlusions. (2) Possible thresholds of the significant parameters were assessed, balancing high sensitivity (maximum number of outcomes in the full collapse group) with having the smallest full collapse group possible. (3) Combinations of thresholds of the significant parameters were assessed in a similar fashion as in step 2. In cases with one parameter was missing, the other parameter was used alone. (4) The risk of the outcome in the traditional and new near-occlusion groups was compared with conventional ≥ 50% stenosis as reference in both unadjusted and multivariable analyses.

Where appropriate, we used 95% confidence interval (95%CI), mean, median, standard deviation (SD), interquartile range (IQR), 2-sided *χ*^2^-test, one-way ANOVA, Mann–Whitney test, and Kruskal–Wallis test. Reliability of CTA measurements was assessed with two-way mixed interclass correlation (ICC) for absolute agreement. The outcome was assessed with Kaplan–Meier (with log rank test) and Cox regression, using revascularization and death as censor. Two multivariable models were assessed: one adjusting for age & sex, the other included baseline parameters and step-wise removing the co-variate with the highest *p*-value until all variables had *p* < 0.05. Two risk markers have been suggested in this context: age and type of presenting event [14]. Age was assessed in a dedicated multivariable analysis. Type of presenting event could not be assessed in this fashion as no case with retinal presenting event (amaurosis fugax or RAO) reached the outcome. Thus, type of presenting event is reasonably an important factor, but not assessable in this study. Statistical significance was defined as *p* < 0.05 and IBM SPSS 26.0 was used in the calculations.

## Results

**Fig. 2:**
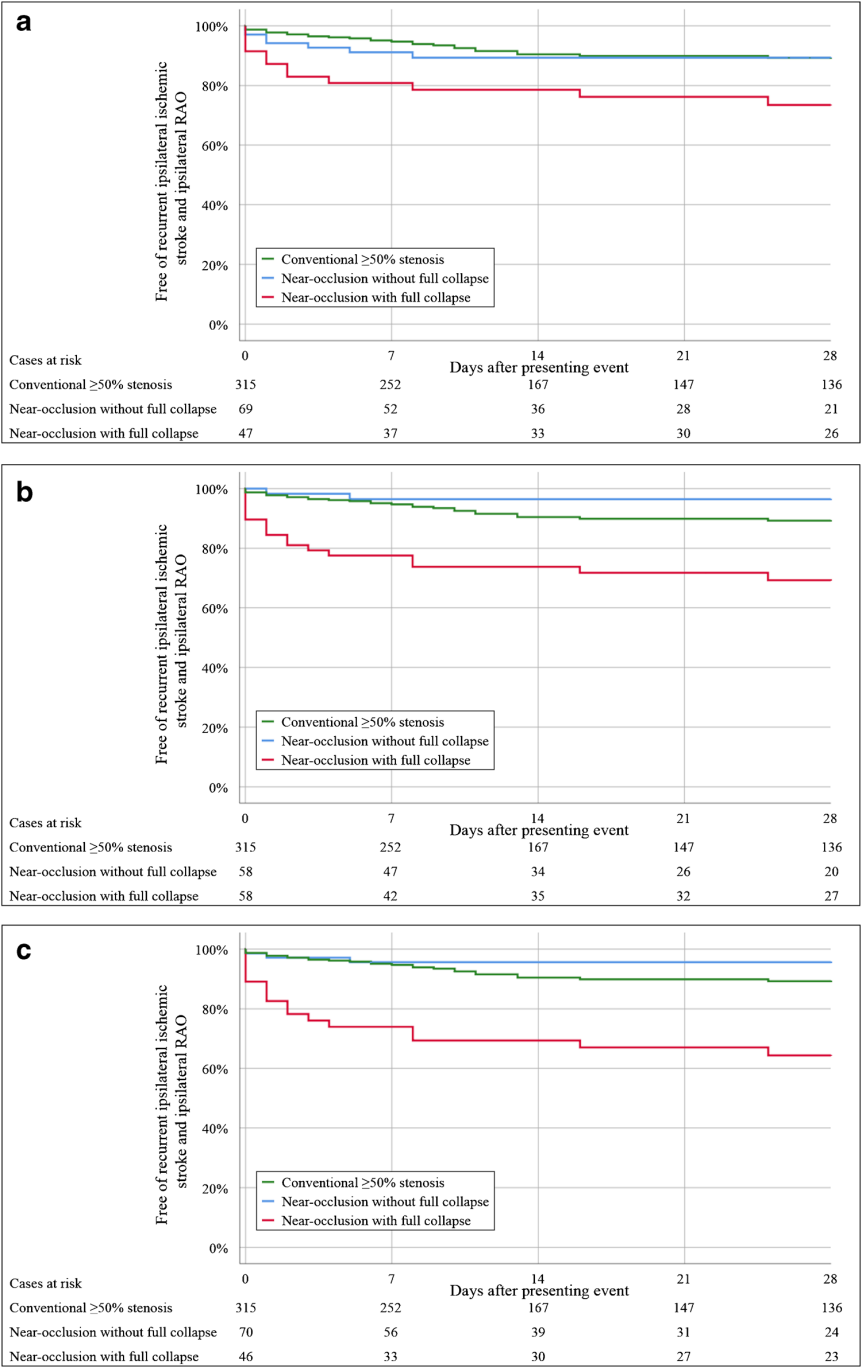
Kaplan–Meier curves of three approaches to define near-occlusion with full collapse. Tests for statistical significance presented in Tables 3 and 5. **a** Traditional approach. **b** Distal ICA diameter ≤2.4 mm. **c** Distal ICA diameter ≤ 2.0 mm and/or ICA ratio ≤ 0.42. The outcome was preoperative recurrent ipsilateral ischemic stroke or ipsilateral retinal artery occlusion

There were 19 outcome events (ipsilateral ischemic stroke or ipsilateral RAO) among the 116 near-occlusions. By traditional definition, the 28-day risk of the outcome was higher in near-occlusion with full collapse (27%) than near-occlusion without full collapse (11%, *p* = 0.047, Table 3, Fig. 2a). Distal ICA diameter, ICA ratio, and ECA ratio were associated with the outcome among near-occlusions (Table 4). Assessing thresholds < 18 outcomes could reasonably be included in a full collapse group as ≥ 18 outcomes would require ≥ 69% of cases in the full collapse group. Several single parameter thresholds had similar overall performance (Table 3). Combining distal ICA diameter ≤ 2.0 mm and/or ICA ratio ≤ 0.42 was both sensitive and with a small full collapse group (Fig. 2c). Other combinations produced similar results, but only this combination made an improvement in either sensitivity or full collapse group size compared to single parameters (data not shown).

**Table 3:** Number of outcomes and Kaplan–Meier curve–based risk of the outcome among near-occlusions at different time intervals after presenting event. Different definitions of full collapse are presented. Conventional ≥ 50% stenosis group presented for general comparison. The outcome was preoperative recurrent ipsilateral ischemic stroke or ipsilateral retinal artery occlusion

Definition of full collapse	Full collapse	Cases (% of near-occlusions)	2 days		7 days		14 days		28 days		*p*^a^
			*n*	% Risk (95%CI)	*n*	% Risk (95%CI)	*n*	% Risk (95%CI)	*n*	% Risk (95%CI)	
Traditional, by appearance	No	69 (59)	4	6 (0–11)	6	9 (2–15)	7	11 (3–18)	7	11 (3–18)	0.047
	Yes	47 (41)	8	17 (6–28)	9	19 (8–30)	10	21 (10–33)	12	27 (14–39)	
Distal ICA diameter ≤ 2.4 mm	No	58 (50)	1	2 (0–5)	2	4 (0–8)	2	4 (0–8)	2	4 (0–8)	< 0.001
	Yes	58 (50)	11	19 (9–29)	13	22 (12–33)	15	26 (15–38)	17	31 (18–43)	
Distal ICA diameter ≤ 2.0 mm	No	71 (61)	3	4 (0–9)	4	6 (0–11)	4	6 (0–11)	4	6 (0–11)	< 0.001
	Yes	45 (39)	9	20 (8–32)	11	24 (12–37)	13	29 (16–43)	15	34 (20–48)	
ICA ratio ≤ 0.47	No	68 (61)	2	3 (0–7)	4	6 (0–12)	4	6 (0–12)	4	6 (0–12)	< 0.001
	Yes	44 (39)	10	23 (10–35)	11	25 (12–38)	13	30 (16–43)	15	35 (21–50)	
ECA ratio ≤ 0.96	No	43 (37)	2	5 (0–11)	4	10 (1–18)	4	10 (1–18)	4	10 (1–18)	< 0.001
	Yes	73 (63)	10	14 (6–22)	11	15 (7–23)	13	18 (9–27)	15	22 (12–32)	
Distal ICA diameter ≤ 2.0 mm and/or ICA ratio ≤ 0.42	No	70 (60)	2	3 (0–7)	3	4 (0–9)	3	4 (0–9)	3	4 (0–9)	< 0.001
	Yes	46 (40)	10	22 (10–34)	12	26 (13–39)	14	31 (17–44)	16	36 (22–50)	
Conventional ≥ 50% stenosis		315 (NA)	9	3 (1–5)	16	5 (3–8)	25	10 (6–13)	27	11 (7–15)	NA

* 95%CI* 95% confidence interval, *ECA* external carotid artery, *ICA* internal carotid artery, *NA* not applicable

^a^Log rank test between the two near-occlusion groups, i.e., conventional ≥ 50% stenosis group not included in these calculations

**Table 4:** Cox regression of the hazard ratio of the outcome among near-occlusions among the five candidate parameters for novel definitions of full collapse. The outcome was preoperative recurrent ipsilateral ischemic stroke or ipsilateral retinal artery occlusion within 28 days of presenting event

Near-occlusion parameter	HR (95%CI)	*p*
Stenosis diameter (per 1 mm increment)	0.44 (0.07–2.53)	0.35
Distal ICA diameter (per 1 mm increment)	0.51 (0.34–0.77)	0.001
ICA ratio (per 0.1 increment)	0.76 (0.64–0.91)	0.03
ECA ratio (per 0.1 increment)	0.86 (0.77–0.96)	0.009
Stenosis PSV (per 1 m/s increment)	1.11 (0.80–1.56)	0.53

*ECA* external carotid artery, *HR* hazard ratio, *ICA* internal carotid artery, *PSV* peak systolic velocity

Multivariable analyses were conducted on the traditional and two of the new full collapse definitions (Table 5). With all three definitions, full collapse had a higher risk than conventional ≥ 50% stenosis in both unadjusted and age & sex–adjusted analyses. The two novel approaches tended to have higher HR for full collapse (3.6–4.4) than the traditional approach (2.9–3.0), but with overlapping 95%CIs. Cases without full collapse showed a weak tendency to have a lower risk than conventional ≥ 50% stenosis with the novel definitions, but not with the traditional definition. In the step-wise removal multivariable analyses, no other variable than degree of stenosis remained at *p* < 0.05 for all three combinations. Age was not associated with the outcome: unadjusted HR 1.1 (95%CI 0.8–1.6, *p* = 0.57) and adjusted for degree of stenosis HR 1.2 (95%CI 0.8–1.7, *p* = 0.40) per 10-year increment, when defining full collapse as distal ICA diameter ≤ 2.0 mm and/or ICA ratio ≤ 0.42.

**Table 5:** Cox regression of the risk of the outcome in the traditional and two novel definitions of full collapse. Unadjusted and age & sex–adjusted models. Conventional ≥ 50% stenosis was used as reference. The outcome was preoperative recurrent ipsilateral ischemic stroke or ipsilateral retinal artery occlusion within 28 days of presenting event

Definition of full collapse	Full collapse	Unadjusted HR (95%CI)	p	Age & sex–adjusted HR (95%CI)	*p*
Traditional, by appearance	No	1.2 (0.5–2.8)	0.66	1.3 (0.5–2.9)	0.59
	Yes	2.9 (1.5–5.7)	0.002	3.0 (1.5–6.0)	0.002
Distal ICA diameter ≤ 2.4 mm	No	0.4 (0.1–1.6)	0.20	0.4 (0.1–1.7)	0.21
	Yes	3.6 (1.9–6.5)	< 0.001	3.7 (2.0–6.9)	< 0.001
Distal ICA diameter ≤ 2.0 mm and/or ICA ratio ≤ 0.42	No	0.5 (0.1–1.6)	0.24	0.5 (0.2–1.7)	0.27
	Yes	4.2 (2.3–7.8)	< 0.001	4.4 (2.3–8.2)	< 0.001

*HR* hazard ratio, *ICA* internal carotid artery

None of the near-occlusion cases with missing data of stenosis diameter or ICA ratio reached the outcome. However, 58% of near-occlusions with the outcome lacked stenosis PSV data, compared to 21% of near-occlusion without the outcome (*p* = 0.002). All but one missing PSV data was due to no ultrasound exam, the exception was due to echo shadow. Median time between presenting event and the outcome tended to be longer for those examined with ultrasound (3, IQR 1–14, days) than those not examined with ultrasound (1, IQR 0–3, days), *p* = 0.15.

Of the 315 conventional ≥ 50% stenoses, 2 (< 1%) had an ECA ratio ≤ 0.96, but none had distal ICA ≤ 2.4 mm or ICA ratio ≤ 0.47.

Intra-rater ICC was excellent for stenosis diameter (0.99; 95%CI 0.98–0.99), distal ICA diameter (0.98; 95%CI 0.97–0.99), and ICA ratio (0.99; 95%CI 0.98–0.99). Inter-rater ICC was very good for ECA ratio (0.93; 95%CI 0.90–0.96).

## Discussion

The main finding of this study was that novel measurement–based definitions of near-occlusion with full collapse seem to provide better prognostic outcomes than the traditional appearance-based approach.

Near-occlusion was defined in NASCET by necessity because the reduction of distal ICA diameter in near-occlusions would lead to stenosis underestimation if the percent comparison was applied [1–3]. Near-occlusion has been, and likely still is, misunderstood by many [2]. Although often described as rare, near-occlusion constitutes 30% of ≥ 50% symptomatic stenosis [15]. Many omit near-occlusion without full collapse [16], even though 94% of near-occlusions in NASCET and ECST were without full collapse [3]. Defining full collapse was initially not done for prognosis, but was rather descriptive and highlighted that not all near-occlusions have a threadlike appearance [1–3]. As symptomatic near-occlusion with full collapse has been shown to have a worse short-term prognosis than those without full collapse [6, 7], a prognostic-based definition is reasonable. Also, “threadlike appearance” is difficult to apply consistently, examples of different applications in the same material exist [3, 17]. By using measurements, better reliability is likely, although that is beyond the scope of this study to assess. The novel definitions only tended to have better prognostic outcome than the traditional definition (not clearly superior), the reliability aspects alone is sufficient to favor the use of a measurement-based approach. As there was no overlap between these new full collapse definitions and conventional ≥ 50% stenosis (except for ECA ratio), the definitions can be applied to all carotid stenosis without risking mistaking a conventional stenosis for near-occlusion with full collapse.

Of the three significant parameters, one was an absolute measurement (distal ICA diameter) and two were relative (ICA ratio, ECA ration), which have different pros and cons: Absolute measurements can vary with study quality, windowing, and handling of the fuzzy edge. While intra-rater reliability was very good, it is not necessarily transferable to raters in routine practice. Relative measurements require a relevant comparison and the comparison can sometimes be misleading, such as bilateral near-occlusion affecting ICA ratio and ECA size vary between patients. Stenosis diameter was not associated with the outcome among near-occlusions, but this could be false-negative due to the use of CTA as stenosis diameter similar to voxel size make assessment difficult. A conventional angiography-based study might yield a positive association. The non-association between stenosis PSV and outcome was possibly due to lacking data. Further study into the prognostic potential of carotid ultrasound in near-occlusion should reasonably include the assessment of distal velocity [18].

Near-occlusion without full collapse had no substantial benefit with CEA in NASCET and ECST due to a relatively low long-term risk of stroke in the medical arm [1–5]. This seems to be applicable to short-term prognosis as well, especially with the new definition. Although our definitions make separation against full collapse easy, a diagnostic challenge remains for separation from conventional stenosis [13, 16]. Further diagnostic work is warranted, which can include both ultrasound and phase contrast approaches [18, 19].

We found that for all symptomatic ≥ 50% stenosis, only degree of stenosis (near-occlusion with full collapse) was associated with the outcome. Higher age and cerebral (not retinal) presenting event have been suggested, but without assessment of near-occlusion [14]. We found no association with age. However, when previously suggested in a study with 377 cases, age was not strongly associated with the outcome (*p* = 0.02). We showed a strong association for full collapse (*p* < 0.001) with similar number of cases included (*n* = 430), suggesting degree of stenosis being more important than age [14].

Our study had several weaknesses. The use of medical treatment could not be assessed as it was gathered with different definitions in the underlying studies [6, 7]. However, dual antiplatelet medication was rarely used [6, 7]. Both of the underlying studies had selection to CTA, albeit it became more standard practice over the 8 years of inclusion [6, 7]. Reliability of ultrasound with test–retest approach was not performed. With the use of adjustable thresholds and limited number of outcomes, some degree of model fitting can be suspected, validation is warranted. Main strengths were the combined data from relevant studies with analyses based on individual data, and reassessment of CTA measurements for a uniform analysis.

In summary, defining near-occlusion with full collapse as distal ICA diameter ≤ 2.0 mm and/or ICA ratio ≤ 0.42 seems to yield better prognostic discrimination than the traditional appearance-based definition. This novel definition can be used in prognostic and treatment studies of near-occlusion with full collapse.

## Data Availability

Data is available on reasonable request.
